# Increase in Lower Limb Strength after Multimodal Pain Management in Patients with Low Back Pain

**DOI:** 10.3390/medicina58070837

**Published:** 2022-06-22

**Authors:** Moritz Kaiser, Sara Brambrink, Achim Benditz, Leonard Achenbach, Matthias Gehentges, Matthias Alexander König

**Affiliations:** 1 Department of Orthopedic Surgery, Regensburg University Medical Center, 93077 Bad Abbach, Germany; bram_sara@yahoo.de (S.B.); matthias.gehentges@web.de (M.G.); matthias.a.koenig@gmail.com (M.A.K.); 2 Department of Orthopedic Surgery, König-Ludwig-Haus, Julius-Maximilians-University Würzburg, 97074 Würzburg, Germany; leonardachenbach@gmail.com

**Keywords:** multimodal pain management, low back pain, muscle strength, dynamometer

## Abstract

*Background and Objectives*: The aim of the present study was to evaluate the efficacy of a multimodal pain therapy (MPM) regarding the objective parameter muscle strength of segment-dependent lower limb muscle groups before and after such a treatment. *Materials and Methods*: 52 patients with a history of low back pain and/or leg pain received standardized multimodal pain management. Strength of segment indicating lower limb muscles were assessed for each patient before and after ten days of treatment by handheld dynamometry. *Results*: Overall strength increased significantly from 23.6 kg ± 6.6 prior to treatment to 25.4 ± 7.3 after treatment, *p* ≤ 0.001. All muscle groups significantly increased in strength with exception of great toe extensors. *Conclusions*: Despite lower basic strength values at the beginning of treatment, all investigated muscle groups, except for the great toe extensors, showed a significant increase of overall strength after completion of the multimodal pain management concept. Increased overall strength could help with avoiding further need of medical care by supporting patients’ autonomy in daily life activities, as well as maintaining working abilities. Thus, our study is the first to show a significant positive influence on lower limb strength in patients with low back pain after a conservative MPM program.

## 1. Introduction

Chronic low back pain (LBP) is of increasing socio-economic importance [1,2]. In 2016, it was one of the five leading causes of years lived with a disability [3]. Furthermore, a prevalence of 1 in 5 in Germany, and 1 in 10 in the United States of America was reported [4,5]. Low back pain, and chronic back pain, is associated with a higher Body Mass Index (BMI) and old age [6,7]. In addition, pain in general is associated with higher immobilization and reduced strength to master daily life activities or the professional life [2]. To avoid persistent need of medical care and to maintain patients’ autonomy, the treatment of this disease is an important issue.

Chronic low back pain is often described in the literature as mixed pain. This means that most patients have both neuropathic and nociceptive pain components [8].

Unless there is an absolute surgical indication such as cauda equina syndrome (CES), conservative therapy should then be carried out first. Since, as mentioned before, chronic low back pain is normally a mixed pain, it should be addressed in a multidisciplinary approach. Different treatment options are described in the literature for the non-surgical treatment [9,10]. In this context, spinal injection therapy has an important role in the treatment of LBP. In addition, it has already been shown that lumbar injection therapy is an effective method for nerve root irritations [11,12].

In combination with accompanying treatments such as physical therapy and psychological counselling as a multimodal analgesia management, injection therapy can be used to control chronic pain symptoms and avoid surgery with accompanying treatments such as physical therapy and psychological counselling [13,14].

Up to now, the visual analogue scale (VAS) or the numeric rating scale (NRS) have mostly been used in the literature as indicators for successful therapy [14]. However, these scales are strictly subjective parameters that can be influenced by a variety of confounders. Patient dependent criteria have mostly been collected through a questionnaire [14,15,16,17]. Interestingly, up to now, no data has been available yet concerning the influence of multimodal pain management (MPM) on muscle strength.

The aim of the present study was to evaluate the efficacy of a multimodal pain therapy regarding the objective parameter muscle strength of segment-dependent lower limb muscle groups before and after such a treatment.

## 2. Material and Methods

### 2.1. Study Population

This prospective study was approved by the local ethics committee on 21 March 2018 (Nr. 18-931-101). Written informed consent was given by all patients participating in this study. Patients were included in the study if they were indicated for inpatient, multimodal pain management in a single center (Department of Orthopedic Surgery, Regensburg University Medical Center, Bad Abbach, Germany) from June 2018 to April 2019 and met the following inclusion criteria: age between 18 and 80 years, radicular pain originating from a specific nerve root or clearly attributable muscle strength impairment of the lower limb without surgical indication, and a pain level of at least 5 on the numeric rating scale.

Exclusion criteria were tumors with spinal implication, congenital spinal malformations, former spinal surgery, and rheumatic or inflammatory spinal diseases. Demographic data are shown in Table 1.

### 2.2. Treatment Algorithm

Inpatient care was provided for each patient for a total of 10 days. The concept consisted mainly of lumbar spinal nerve root analgesia (LSPA) in the affected region, using a freehand technique, which has already been described elsewhere [18]. The injections were administered twice a day in the morning and at noon. In addition, the patient received a bilateral facet joint infiltration at the level L4 to S1 and an epidural injection, as well a nerve root block. Undiluted Mepivacaine 1% was used and 10 mL were applied as LSPA. For the lumbar epidural injection, 8 mg Dexamethasone were combined with sterile saline solution. Again, 10 mL were applied. Facet joint injections contained 8 ml undiluted Mepivacaine 1% and 8 mg Dexamethasone. For nerve root blocks, 4 ml Mepivacaine 1% and 4 mg Dexamethasone were injected. The aforementioned injections were accompanied by physiotherapeutic and sports medical exercises, which included isometric strengthening of the back muscles on specific training equipment. A specific training program for lower limb muscles was not carried out. Additionally, the patients received proprioceptive training, electrotherapy, thermotherapy, instruction in progressive muscle relaxation, and if indicated, a psychotherapeutic program, which was embedded in a group therapy.

### 2.3. Measurement

A hand-held dynamometer (microFET2, Hoggan Health Industries, Salt Lake City, Utah, USA) was used to measure strength. All values were documented in kilogram. The measurements were performed on two different days. The first measurement took place on the day of admission to determine the basic strength values, and the second on the day of discharge. Each measurement was performed independently by two examiners for each assessment. Both examiners received instruction and training in the use of the measurement device prior to the study.

**Figure 1:**
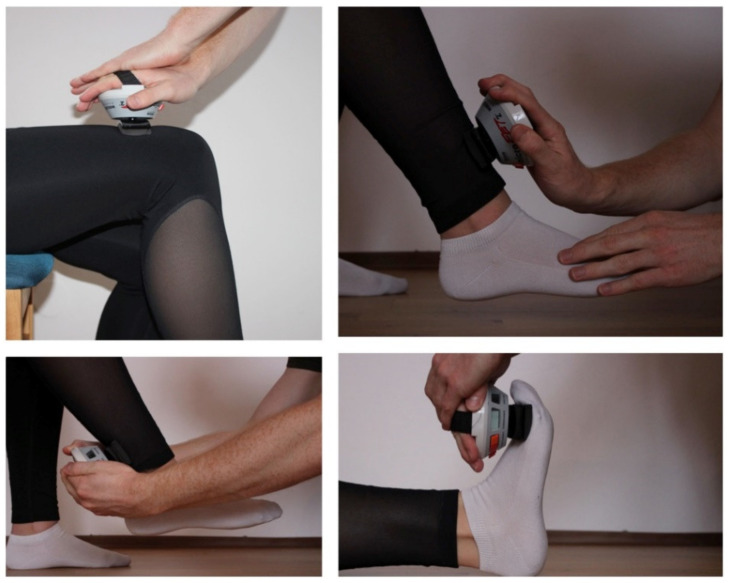
Different testing positions.

The contact surfaces were marked with a skin-friendly pencil to ensure equal measurement points. The marked areas were repainted by the patient himself, if necessary, (e.g., after personal hygiene acts). The contact surfaces were chosen in a manner that allowed for comfortable measurement with the highest achievable leverage. The best testing position for each muscle group was described by Mentiplay [19] While in a sitting position, the following movements were then carried out, one after the other, against the resistance of the dynamometer using the “break method” as described by Burns and Spanier [20]: hip flexion, knee extension, knee flexion. Then, the patient was moved into a lying position. Starting from the neutral joint position, the following movements were performed: foot extension, foot flexion, big toe extension. Each measurement was carried out bilaterally and all movements were measured in every patient. Three different types of transducer pads were available for the device in order to achieve a proper skin contact, a flat one, a curved one and a small flat one for Toes. Figure 1 illustrates exemplarily the testing positions for hip flexion, knee flexion, knee extension and ankle flexion.

To record the patients’ pain, a numerical rating scale (NRS) from 0 to 10 was used. The patients were interviewed before each measurement regarding the pain level. The strength values were documented immediately in standardized, electronic form. In addition, the descriptive data of every individual was collected (Table 1 and Table 2).

### 2.4. Statistical Analysis

For statistical analysis, continuous data are presented as mean values and standard deviation. Group comparisons were performed by two-sided *t*-tests for dependent variables. Absolute and relative frequencies were given for categorical data. Inter-observer agreement was assessed using the intraclass correlation coefficient (ICC). The following values were determined according to Koo et al. [21]: less than 0.5 poor, between 0.5 and 0.75 moderate, between 0.75 and 0.9 good, and greater than 0.90 excellent reliability. Differences of *p* < 0.05 were considered statistically significant. IBM SPSS Statistics 25 (SPSS Inc, Chicago, IL, USA) was used for analysis.

## 3. Results

In total, 52 Patients were included analyzed with a mean age of 63.2 years (±12.1), 25 male and 27 female. Patients were treated in-hospital for 9.19 days (±0.56) on average. The painful side was right in 12 patients, left in 15 patients, and both in 25 patients.

Overall Interrater Reliability was excellent with ICC = 0.94 for measurements at the day of admission and excellent with ICC = 0.96 for the second measurements at discharge.

NRS decreased significantly for both back and leg pain from 5.9 (±2.2) to 3.3 (±2.2) and 4.5 (±2.8) to 2.5 (±2.0), respectively, *p* < 0.001.

Infiltration of the facet joints were administered 45 times (86.5%), epidural injections 47 times (90.4%), and nerve root blocks 19 times (36.5%).

### 3.1. Strength Development

Hip flexors strength increased significantly (*p* = 0.013) by 7.2% from the first day to discharge.

For the knee extensors, the power of the muscle group increased significantly by 12.6% (*p* < 0.001). 

**Figure 2:**
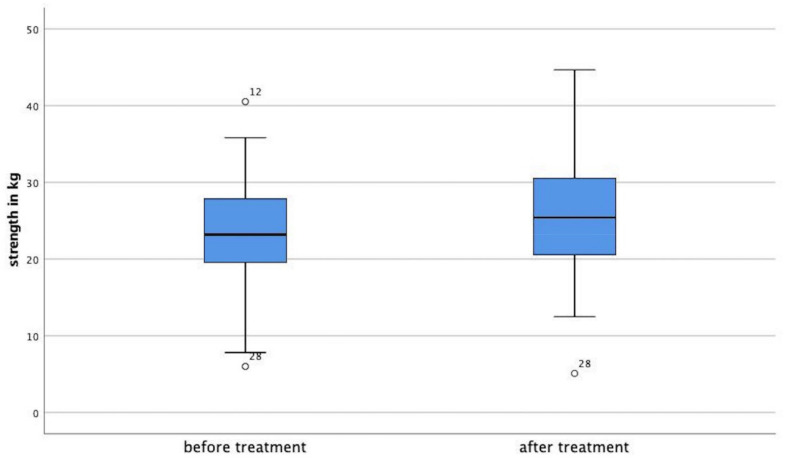
Overall mean strength in kg before and after treatment. Outliers are marked as circles.

Knee flexors showed an increased rate of 7.4%, (*p* < 0.001). Ankle extensor strength increased by 4.75% (*p* = 0.041). Ankle flexor strength increased by 9.5% (*p* < 0.001). For great toe extensors, a reduction of strength was noted by −4.0%, which was statistically not significant, *p* = 0.103. Mean values for strength development are shown in Table 2. Figure 2 shows Boxplots prior to treatment and after for mean overall strength.

### 3.2. Side-to-Side Differences

An overall significant side-to-side difference prior to treatment, considering all measurements on one side, was not noticed, *p* = 0.294. Overall strength on the right side was 23.96 kg ± 7.4, and on the left was 23.24 kg ± 6.7. After treatment, a significant side-to-side difference was recognized, *p* = 0.039. On the right side, overall strength was 25.87 kg ± 7.6, on the left side, it was 24.95 kg ± 7.3.

No adverse events were found in the study.

## 4. Discussion

The most important finding of this study was that lower limb strength increased significantly within a few days of non-operative treatment in patients with low back pain.

Up to now, objective data regarding the course of segment-dependent muscle strength in patients undergoing MPM are missing. Hence, the primary goal of this study was to objectify the influence of multimodal pain management with regard to the muscle strength development of lower limb muscles using a hand-held dynamometer.

There are several limitations of this study. First, the strength values were assessed only twice, at the start of the injection therapy and at its end. Therefore, our records do not contain any information about the course of strength development over the entire duration of therapy. Second, a handheld dynamometer has been used in the current study. However, a free hand technique cannot be as accurate as a fixed system due to imbalances in motion control of the raters and variances due to the raters themselves. Third, there is no comparison group, especially comparing injections to other treatment options like analgesia or physiotherapy alone in a multimodal analgesia concept. This might limit the interpretation of the data. In addition, the study group is very limited. Bigger study groups are needed to confirm the findings of this investigation. Since there were only two measurements in a very short period, it is not possible to predict a long-term result and how long the effect of MPM on muscle strength might last.

Researchers have to be aware when comparing different testers. As Wadsworth and Wikholm showed in their studies, intertester reliability is reduced when the strength of the subject is superior to the one of the examiner [22,23]. Third, the present study does not contain any information about the patient’s dominant side. Compared to the results in the literature, the presented measuring method could have an unintentional influence by the examiner himself, based on different force and gender as well as, of course, levers of the examiner [24,25].

In the present study, the interrater reliability results for both before treatment and after treatment proved to be excellent with values of 0.94 and 0.96, respectively. These findings are comparable to literature, where a total interrater reliability of 0.94 using the same device can be found [26]. Nonetheless, there are also inhomogeneous results, ranging from moderate to excellent interrater reliability between assessors testing muscle strength with a hand-held device [27,28,29].

In general, the comparison of the available data with the literature does not appear to be unambiguous due to inhomogeneous measurement methods. For hip flexion, the values of 24.8 kg ± 9.7 were found after treatment, which is a good average of the literature. A study conducted by Andrews et al., used a different testing position and device strength levels for hip flexion in asymptomatic, elderly adults, and found values of 18.8 kg, while Lasse Ishøi et al., reported a peak force of hip flexion in healthy young adults of up to 321 N (32.7 kg) with a similar testing setup [30,31].

In 2000, Stoll et al., published higher maximal isometric muscle strength in hip flexion values of 25.1 kp (1 kp = 9.81 N) to 38.0 kp in healthy adults [32]. The increased hip flexion muscle strength in healthy adults compared to patients with low back pain might be the result of pain-free range of motion. In the present study, knee extension and flexion force was 30.3 kg ± 12.09 and 21.75 kg ± 7.31, respectively, both after treatment. These findings are more or less in line with Stoll et al., which found slightly more muscle strength in these groups [32]. As they investigated healthy participants, the results in this study suggest a normalization of high pain levels resulting in low strength levels to the ones of asymptomatic adults due to MPM. Also, a significant rise in knee strength was found after treatment, suggesting patient relief from pain-related functional restrictions. Lee et al., reported that patients with a history of low back pain have significantly lower strength levels in total knee strength than a control group [28].

For ankle dorsal extension and plantar flexion, a significant increase was also found, showing a considerably higher base value for the plantar flexors of about 14% in comparison to the dorsal extensor, which increased even after treatment to about a 21% difference. Mentiplay et al., could show higher levels of baseline values, ranging from about 21 kg and 31 kg for dorsal extensors and about 48 kg and 51 kg for plantar flexors [19]. This indicates an even higher plantar flexion and dorsal extension strength ratio, which highlights the increasing strength ratio after treatment in the present study.

The results for great toe extensors in this study slightly decreased over the treatment period. However, clinical testing of the extension force of the big toe is difficult due to the small contact surface on the one hand and the rather low extension force of the big toe compared to the force of the examiner’s hands on the other. Interestingly, the extension force of the big toe has rarely been studied in literature, despite the fact that the extensor hallucis longus is the segment-dependent muscle of the L5 nerve root. In 1995, Jönsson et al., reported an improvement in the function of the extensor hallucis longus after surgical treatment of disc herniation and affection of the L5 nerve root, but the classification of the force ratios was only categorical and not absolute [33]. Further studies also did not present absolute data obtained by dynamometry, such as Hara et al., in 2011, who assessed extensor muscle strength by placing the middle finger of the investigator on the toe [34]. Riandini et al., used a Hoggan Microfet 3 but presented their data only relative [35]. The discrepancy in measurement should be the content of further research to compare these findings, since L5 nerve root irritations are common.

After treatment, the patients showed a significant side-to-side-difference, which was not the case prior to the multimodal pain management. According to Lanshammer et al., normally existing side-to-side differences reoccur, which emphasizes the positive effect of the multimodal pain therapy [36]. However, side differences are controversially discussed in literature. Andrews et al., did not see significant side-to-side differences in the lower limbs in asymptomatic patients [30]. The clinical relevance of the study is based on the fact that patient with low back Pain seem to highly benefit from a MPM. Handheld dynamometry is a useful instrument to monitor the success.

## 5. Conclusions

Despite lower basic strength values at the beginning of treatment, all investigated muscle groups, with the exception of the great toe extensors, showed a significant increase of overall strength after completion of the multimodal pain management concept. Increased overall strength could help with avoiding further need of medical care by supporting patients’ autonomy in daily life activities, as well as maintaining working abilities. Thus, to our best knowledge, the present study is the first to show a significant positive influence on lower limb strength in patients with low back pain after a conservative MPM program. Altough, our findings are of limited value (low level of evidence), and it is unknown whether these effects are sustained in the long term.

## Figures and Tables

**Table 1:** Demographic data.

*n*	52
Gender (male/female)	25/27
Age (years ± SD)	63.2 ± 12.1
BMI (kg/m^2^ ± SD)	30.6 ± 6.3
Painful side (right/left/both)	12/15/25
Treatment days (±SD)	9.19 ± 0.56

BMI—Body Mass Index, SD—standard deviation.

**Table 2:** Strength development.

Muscle Group	M1 in kg	M2 in kg	*p*-Values
HF	23.17 ± 8.61	24.84 ± 9.70	*p* = 0.013
KE	26.92 +/− 10.26	30.30 ± 12.09	*p* ≤ 0.001
KF	20.26 ± 7.03	21.75 ± 7.31	*p* ≤ 0.001
ADE	29.75 ± 9.76	30.90 ± 10.29	*p* = 0.041
APF	34.02 ± 7.13	37.24 ± 7.3	*p* ≤ 0.001
GTE	7.71 ± 2.64	7.40 ± 2.31	*p* = 0.103

M1—Measurement 1, M2—Measurement 2, HF—hip flexors, KE—knee extensors, KF—knee flexors, AE—ankle dorsal extensors, AF—ankle plantar flexors, GTE—great toe extensors.

## Data Availability

The data that support the findings of this study are available from the corresponding author upon reasonable request.
